# Successful Treatment of Post‐pump Chorea With Tiapride in an Adult Cardiac Surgery Patient: A Case Report

**DOI:** 10.1155/crcc/5776661

**Published:** 2026-09-10

**Authors:** Nicolas Kaiser, Matthias C. Borutta, Martin Regensburger, Larissa Herbst, Lena Rühl, Timo Seitz, Stefan Gerner, Mario Schiffer, Carsten Willam, Kosmas Macha, Karl Bihlmaier

**Affiliations:** ^1^ Department of Nephrology and Hypertension, Friedrich–Alexander-University Erlangen-Nürnberg (FAU), University Hospital Erlangen, Erlangen, Germany, uk-erlangen.de; ^2^ Department of Neurology, Friedrich–Alexander-University Erlangen-Nürnberg (FAU), University Hospital Erlangen, Erlangen, Germany, uk-erlangen.de; ^3^ Department of Molecular Neurology, Friedrich–Alexander-University Erlangen-Nürnberg (FAU), University Hospital Erlangen, Erlangen, Germany, uk-erlangen.de; ^4^ Department of Cardiac Surgery, Friedrich–Alexander-University Erlangen-Nürnberg (FAU), University Hospital Erlangen, Erlangen, Germany, uk-erlangen.de

**Keywords:** cardiac surgery, cardiopulmonary bypass, choreiform movements, post-pump chorea, tiapride

## Abstract

Postpump chorea is a rare hyperkinetic movement disorder following cardiac surgery with cardiopulmonary bypass. Although initially described in pediatric patients, postpump chorea is increasingly recognized in adults. We report the case of a 65‐year‐old woman who developed severe choreiform movements approximately 2 weeks after cardiac surgery. Extensive diagnostic workup including cerebrospinal fluid analysis revealed no alternative etiology. Notably, hypothermia had only been mild during cardiac surgery, and there were no characteristic basal‐ganglia MRI abnormalities. Treatment with tiapride, a selective dopamine D2/D3 receptor antagonist with established antichoreic effects was followed by rapid neurological improvement and complete remission of symptoms. This is one of the first reports of tiapride in adult postpump chorea, supporting its role as a potentially useful symptomatic therapeutic option in this rare complication.

## 1. Introduction

Postpump chorea (PPC) is a rare hyperkinetic movement disorder occurring after cardiac surgery with extracorporeal cardiopulmonary bypass (CPB). Although predominantly described in pediatric patients [1], adult cases have been reported with increasing frequency [2, 3]. Identified risk factors include prolonged CPB duration and hypothermia, whereas proposed mechanisms involve microembolic basal ganglia injury and inflammatory processes [1, 4–6]. PPC typically manifests after a latency of several days to 2 weeks following CPB [1, 4]. Treatment is symptomatic and mainly consists of dopamine antagonists and benzodiazepines [4], with variable outcomes. Here, we describe an adult case of PPC with rapid and sustained response to tiapride.

## 2. Case Presentation

### 2.1. Symptoms and Investigations

A 65‐year‐old woman with a pre‐existing diagnosis of mild cognitive impairment underwent combined replacement of the aortic valve, aortic root, and ascending aorta for aortic stenosis and aneurysm. Her medical history included stable mild cognitive impairment for over 15 years. Routine surgery was performed, with a CPB duration of 3 h and 21 min and an aortic cross‐clamp time of 2 h and 29 min. Normothermia was maintained throughout the procedure (minimum temperature 35.3° C).

Postoperatively, the course was complicated by hyperactive and hypoactive delirium treated with haloperidol, followed by aspiration pneumonia requiring re‐intubation on postoperative Day 5. Broad‐spectrum antimicrobial therapy was initiated, and transient fever occurred between postoperative Days 12 and 16. Respiratory function gradually improved; however, extubation was impeded by reduced arousal and the onset of progressive choreiform movements of all body areas (extremities, truncal, cervical, facial, and bucco‐oro‐lingual) on postoperative Day 14. There was no startle trigger and the patient was not able to voluntarily suppress or modulate the choreiform movements.

Neurological examination revealed severely impaired consciousness with preserved brainstem reflexes and no pyramidal signs. Antagonist therapies (flumazenil, naloxone, and physostigmine) and empirical treatments including levetiracetam, biperiden, and amantadine were ineffective. Repeated cranial computed tomography scans did not show any pathologies. Magnetic resonance imaging demonstrated nonspecific supratentorial microbleeds. Repeated cerebrospinal fluid (Table 1) and blood analyses (Table 2) did not show any pathological alterations. Electroencephalography showed an encephalopathic pattern without epileptiform activity and further neurophysiological studies were nondiagnostic.

**Table 1:** Results of cerebrospinal fluid testing. Displayed are the date of the CSF tests, the test parameters, and the reference values.

Post‐surgery day	Test	Result	Norm
12	Protein	129 mg/L	150−450
	Cell count	16/*μ*l	
	Leucocytes	3/*μ*l	< 5
	Erythrocytes	13/*μ*l	
	CMV, VZV, HSV‐1/2, EBV (PCR)	Negative	Negative
	Microscopy (bacteria, fungi)	Negative	Negative

30	Leucocytes	6/*μ*l	< 5
	Erythrocytes	346/*μ*l	
	Glucose	73 mg/dl	
	Lactate	1.64 mmol/l	1, 2−2, 1
	CMV, HSV‐1/2 (PCR)	Negative	Negative
	Microscopy (bacteria, fungi)	Negative	Negative
	Universal bacterial PCR	Negative	Negative
	Universal fungi PCR	Negative	Negative
	Antibasalganglia antibodies (IFA)	Negative	Negative
	Antineuronal antigens (IFA)	Negative	Negative
	Antineuronal antigens (IB)	Negative	Negative

35	Protein	197 mg/l	150−450
	Leucocytes	1/*μ*l	<5
	Erythrocytes	4/*μ*l	
	Glucose	81 mg/dl	
	Lactate	1.69 mmol/L	1.01−2.09
	Oligoclonal bands	Negative	Negative

**Table 2:** Results of blood and urine testing. Displayed are the date of the serum and urine tests, the test parameters, and the reference values.

Postsurgery day	Test	Result	Norm
30	Antineuronal antigens (IFA)	Negative	Negative
	Antineuronal antigens (IB)	Negative	Negative
32	Ammonia	49 *μ*g/dL	19−82

34	Antinuclear antibodies	Negative	Negative
	Cardiolipin antibodies IgG	3.16 GPL U/mL	0−14
	Cardiolipin antibodies IgM	2.16 MPL U/mL	0−7.5
	*β*2 glcoprotein antibodies IgG	2.52 U/mL	0−14
	*β*2 glcoprotein antibodies IgM	2.48 U/mL	0−14
	Lupus anticoagulant	Negative	Negative
	Antistreptolysin	200 IE/mL	< 200
	Coeruloplasmin	0.28 g/L	0.2−0.6
	Copper	0.91 mg/L	0.74−1.22
	Free copper	< 0.10 mg/L	< 0.40
	Urinary copper	37.4 *μ*g/24 h	< 60

### 2.2. Diagnosis, Treatment, and Outcome

After exclusion of alternative diagnoses, PPC was diagnosed based on the characteristic clinical presentation. Given the severe generalized hyperkinetic movements in a critically ill patient with markedly impaired consciousness, a targeted antidopaminergic treatment with a favorable neurological tolerability profile was preferred. Tiapride was selected as first line because of its potentially lower risk of extrapyramidal side effects [7] although no direct comparison studies to VMAT2 inhibitors such as tetrabenazine exist. Tiapride was initiated on postoperative Day 34 and titrated to 200 mg three times daily. Within 3 days, the patient began to follow commands. Over subsequent weeks, choreiform movements progressively subsided. During neurological rehabilitation, cognitive engagement and communication steadily improved. Tiapride was discontinued after several weeks without recurrence of symptoms during follow‐up.

## 3. Discussion

Our patient developed PPC approximately 2 weeks after cardiac surgery with CPB, consistent with the typical latency reported in the literature [4]. Extensive diagnostic evaluation failed to identify infectious, autoimmune, metabolic, or structural causes. Notably, PPC occurred despite maintained normothermia, which contrasts with many previously reported adult cases [2, 3, 8]. A pre‐existing diagnosis of mild cognitive impairment might have led to increased susceptibility for postoperative delirium and potentially also PPC.

Differential diagnoses included infectious or autoimmune encephalitis and neuroleptic malignant syndrome. The latter was considered because of haloperidol exposure and fever but was deemed unlikely given a low cumulative dose, absence of rigidity, and lack of biochemical evidence for muscle serum parameters [9]. Neuroimaging findings were normal or did not account for the severity of clinical symptoms [3, 6].

Therapeutic evidence for PPC remains limited to case reports and small series, and no established treatment algorithm exists. Dopamine receptor antagonists, VMAT2 inhibitors, antiseizure medications, and benzodiazepines have been used with variable success [4, 10], whereas corticosteroid responsiveness has recently been described [6]. To our knowledge, this is the first report of PPC successfully treated with tiapride. Tiapride is regarded as a selective dopamine D2/D3 receptor antagonist with preferential limbic and striatal activity [11]. Given its preferential striatal activity and favorable safety profile, tiapride represents an attractive option in critically ill patients. The close temporal association between initiation of therapy and rapid clinical improvement supports a substantial treatment effect, although spontaneous recovery cannot be entirely excluded. However, severely impaired consciousness is not typically observed in PPC. Its prompt resolution following initiation of tiapride further supports the appropriateness and potential effectiveness of this therapeutic approach.

## 4. Conclusion

This case highlights PPC as a distinct postoperative neurological complication of cardiac surgery with CPB that may even occur in the absence of profound hypothermia or characteristic MRI abnormalities. Thorough exclusion of alternative etiologies is essential. Our findings suggest that tiapride may represent an effective and well‐tolerated treatment option for PPC and warrant further observation in future cases.

**Nomenclature**
CPB: cardiopulmonary bypass
PPC: postpump chorea

## Author Contributions

T.S. was responsible for cardiac surgery and primary postoperative care. N.K., L.H., T.S., M.S., C.W., and K.B. treated the patient during the extended stay in the intensive care unit and performed first neurological diagnostics. M.C.B., M.R., L.R., S.G., and K.M. treated the patient and completed neurological diagnostics. N.K., C.W., and K.B. wrote the first draft of the manuscript. M.C.B., M.R., and K.M. finalized the manuscript. All authors discussed the manuscript and approved the submitted version. The corresponding authors have full access to all of the data in this study and take complete responsibility for the integrity of the data and the accuracy of the data analysis.

## Funding

Open Access funding enabled and organized by Projekt DEAL.

## Disclosure

All authors affirm that this manuscript is an honest, accurate, and transparent account of the study being reported; that no important aspects of the study have been omitted; and that any discrepancies from the study as planned have been explained. All authors have read and approved the final version of the manuscript.

## Ethics Statement

Approval for case reports is not required by the Ethics Committee of the Friedrich–Alexander‐University Erlangen‐Nuremburg.

## Consent

Written informed consent for publication of their clinical details was obtained from the legal representatives of the patient. A copy of the consent form is available for review by the editor of this journal.

## Conflicts of Interest

All authors declare no conflicts of interest.

## Data Availability

The data supporting these findings are available from the corresponding author
